# Enhancement in sustained release of antimicrobial peptide and BMP-2 from degradable three dimensional-printed PLGA scaffold for bone regeneration

**DOI:** 10.1039/c8ra08788a

**Published:** 2019-04-04

**Authors:** Lei Chen, Liping Shao, Fengping Wang, Yifan Huang, Fenghui Gao

**Affiliations:** Department of Joints and Sports Medicine, The First Hospital of Jilin University Changchun PR China; Department of Orthopedic, The First Hospital of Jilin University Changchun PR China; Department of Joints Surgery, The First Hospital of Jilin University Changchun PR China

## Abstract

One of the goals of bone tissue engineering is to create scaffolds with well-defined, inter-connected pores, excellent biocompatibility and osteoinductive ability. Three-dimensional (3D)-printed polymer scaffold coated with bioactive peptide are an effective approach to fabricating ideal bone tissue engineering scaffolds for bone defect repair. However, the current strategy of adding bioactive peptides generally cause degradation to the polymer materials or damage the bioactivity of the biomolecules. Thus, in this study, we used a biomimetic process *via* poly(dopamine) coating to prepare functional 3D PLGA porous scaffolds with immobilized BMP-2 and ponericin G1 that efficiently regulate the osteogenic differentiation of preosteoblasts (MC3T3-E1) and simultaneously inhibit of pathogenic microbes, thereby enhancing biological activity. In this study, we analysed a 3D PLGA porous scaffold by scanning electron microscopy, water contact angle measurements, and materials testing. Subsequently, we examined the adsorption, release and *in vitro* antimicrobial activity of the 3D PLGA. Surface characterization showed that poly(dopamine) surface modification could more efficiently mediate the immobilization of BMP-2 and ponericin G1 onto the scaffold surfaces than physical adsorption, and that ponericin G1-immobilized 3D PLGA scaffolds were able to maintain long-term antibacterial activity. We evaluated the influence on cell adhesion, proliferation and differentiation by culturing MC3T3-E1 cells on different modified 3D PLGA scaffolds *in vitro*. The biological results indicate that MC3T3-E1 cell attachment and proliferation on BMP-2/ponericin G1-immobilized 3D PLGA scaffolds were much higher than those on other groups. Calcium deposition, and gene expression results showed that the osteogenic differentiation of cells was effectively improved by loading the 3D PLGA scaffold with BMP-2 and ponericin G1. In summary, our findings indicated that the polydopamine-assisted surface modification method can be a useful tool for grafting biomolecules onto biodegradable implants, and the dual release of BMP-2 and ponericin G1 can enhance the osteointegration of bone implants and simultaneously inhibit of pathogenic microbes. Therefore, we conclude that the BMP-2/ponericin G1-loaded PLGA 3D scaffolds are versatile and biocompatible scaffolds for bone tissue engineering.

## Abstract 2

One of the goals of bone tissue engineering is to create scaffolds with well-defined, inter-connected pores, excellent biocompatibility and osteoinductive ability.

## Graphical abstract

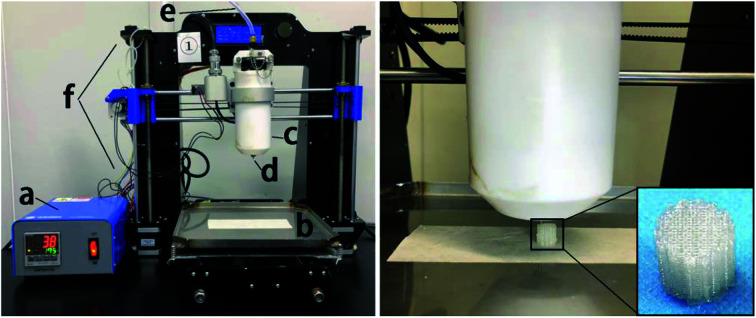

## 1. Introduction

Bone regeneration in bone defect arising from trauma, surgical resection, or skeletal abnormalities is an important consideration. Many approaches have been widely applied to repair bone defects, including autografts, allografts, and xenografts.^1^ However, these therapeutic methods have many associated drawbacks that limit their development, such as limited availability, transfer of pathogens, non-biodegradability, and secondary infections.^2,3^ In recent years, bone tissue engineering has shown great potential for the development of new biomaterials as bone replacement materials and bone regeneration therapy.^4^ Ideally, bone tissue engineering scaffolds should mimic the extracellular matrix (ECM) and regulate cell function. At the same time, scaffolds should be biocompatible, biodegradable, and have the required mechanical strength. To fabricate an ideal bone tissue engineering scaffold, a variety of techniques are available, including electrospinning melt extrusion, emulsion templating, self-assembly, phase separation and three-dimensional (3D) printing.^5–9^ Of these techniques, 3D printing has been extensively investigated as an important technique for the production of bone tissue engineering scaffold because 3D scaffolds can provide more complex, multiscale structures and favorable biological environments for cells than other bone scaffolds.^10^ An important advantage of 3D printing technology is that highly permeable pores can be prepared by computer-aided devices. In the process of scaffolding construction, the fiber direction can be precisely controlled, thus affecting the mechanical strength of the scaffold. At present, various 3D-printed bone scaffolds made of synthetic polymers such as PLA, PCL and PLGA have been widely use utilized as cell-supporting matrices for bone repair.^11–13^

Although 3D printing technology can be used to successfully prepare scaffolds with adjustable morphologies, a significant drawback of this type of 3D polymer bone scaffold, which is widely recognized by researchers, is their hydrophobic surfaces, limited osteoinductive ability and lack of bioactivity, which has limited their application in bone defect repair.^14^ One strategy for solving this problem is performing a surface modification in which a bioactive factor is incorporated into the 3D scaffolds to accelerate bone healing, which has been shown to be effective in numerous studies of the encapsulation of drugs such as growth factors, curcumin and collagen.^15–17^ Among osteogenic-related bioactive factors, the bone morphogenetic protein-2 (BMP-2) is a key factor in bone defect repair and has been widely used in tissue engineering approaches for the repair of bone injuries and bone defects. BMP-2 can improve gene expression during osteogenic differentiation *in vitro* including that of osteopontin, osteocalcin, bone sialoprotein, and alkaline phosphatase (ALP).^18,19^ Previous studies have demonstrated that after incorporating of BMP-2 into polymer scaffolds, the scaffolds exhibit not only adequate mechanical strength, and the required biodegradation rate and morphological structure, but can also effectively deliver the growth factor for actively guiding and accelerating cell attachment, proliferation and differentiation in the scaffolds.^20,21^ However, since BMP-2 is a protein, and proteins have physical and chemical instabilities, excessive processing accelerates BMP-2 inactivation and reduce its effectiveness. Therefore, an effective method must be developed for incorporating BMP-2 in polymer scaffolds.

In recent years, a poly (dopamine) deposition method for incorporating peptides or growth factors has also been widely utilized to improve the regenerative capacity of cells for bone regeneration *in vitro* and *in vivo*.^22–24^ This polydopamine (pDA)-assisted immobilization strategy was inspired by the adhesive mechanism of mussels. Under alkaline conditions, the oxidation of catechol groups forms a pDA layer on various materials, such as polymers and metals. This may offer a simple and effective platform for immobilizing peptides and proteins on polymeric scaffolds. This novel method seems to be relatively simple, stable and effective in immobilizing peptides and proteins on polymer scaffolds, as compared to previous methods such as direct incorporation or simple physical adsorption. Furthermore, many studies have found that factors immobilized *via* pDA maintain high activity, and that the immobilization process dose not change the structure of the material.^25^ A pDA coating can also promote the significant adhesion and proliferation of osteoblasts and the nucleation of hydroxyapatite by its catechol moieties, which can improve the osteointegration of polymer bone scaffolds.^26,27^ Besides osteoinductive ability, an important property of the ideal bone scaffold is its ability to protect damaged bone tissue against microbial growth to prevent infections, as antibiotics are no longer an ideal solution due to their challenges in reaching the target organisms, especially when associated with a medical device. Ponericin G1, an antimicrobial peptide that belongs to the cecropin-like family, is isolated from the venom of the ponerine ant *P. goeldii*.^28^ Besides its excellent antibacterial properties, which include a broad spectrum of activity against bacteria and fungi without affecting most other eukaryotic cells, it has also been reported to be biocompatible with relevant healing cells.^29,30^ Given the above, we speculate that the combined application of a growth factor and antimicrobial peptide can be an effective method for preparing functional polymer bone scaffolds with improved osteoinductive and antibacterial abilities.

**Scheme 1:**
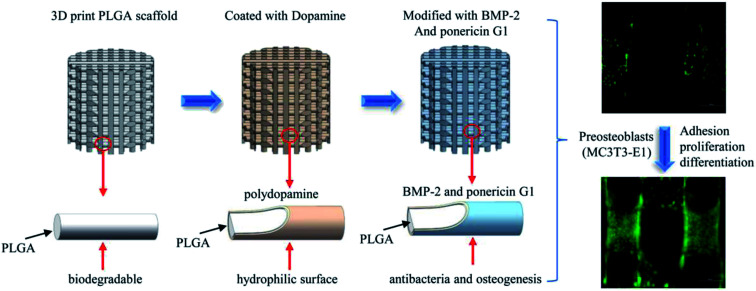
Schematic illustration of sustained release of antimicrobial peptide and BMP-2 from 3D-printed PLGA scaffold for osteogenesis differentiation.

In this study, we report the pDA-assisted immobilization of BMP-2 and ponericin G1 on 3D-printed PLGA scaffolds to enhance the cell adhesion and osteogenic differentiation of mouse preosteoblasts (MC3T3-E1) *in vitro*, as shown in Scheme 1. For this purpose, we fully characterized the physicochemical and mechanical properties of BMP-2/ponericin G1-immobilized 3D PLGA scaffolds using various techniques including scanning electron microscopy (SEM), micro-computed tomography analysis (micro-CT), contact angle measurements, and a materials testing. We explored the combined effects of BMP-2 and ponericin G1 on cell attachment, cell proliferation, and osteogenic differentiation to investigate the effectiveness of functional 3D-printed PLGA bone scaffolds.

## 2. Experimental section

### 2.1 Materials

We purchased poly(l-lactide-*co*-glycolide) (PLGA) (lactide : glycolide (75 : 25), *M*_w_ 150 000 g mol^−1^) from SinoBiomaterials Co., Ltd., ponericin G1 peptide and N-terminal FITC-labeled ponericin G1 peptide from top-peptide Co., Ltd. BMP-2 from UB Biotech and dopamine hydrochloride (98%) from Aldrich. *Staphylococcus aureus* (*S. aureus*) and *Escherichia coli* (*E. coli*) were purchased from ATCC. MC-3T3-E1 cells were obtained from the Cell Culture Centre of Institute of Basic Medical Sciences Chinese Academy of Medical Sciences.

### 2.2 Fabrication of 3D-printed scaffold and surface modification

**Fig. 1:**
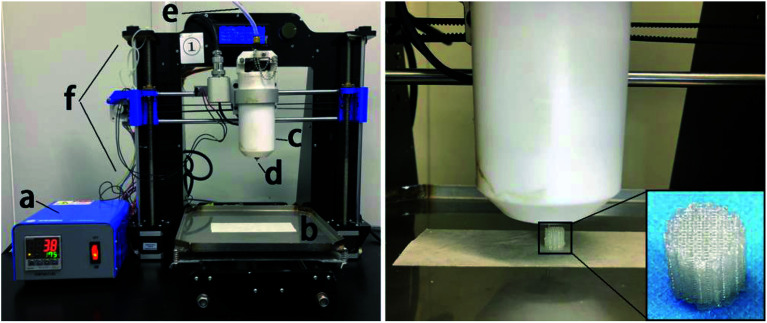
Schematic diagrams of: (a) temperature controller, (b) receiving plate, (c) heating tube, (d) print nozzle, (e) nitrogen, (f) 3D printer and the printing process of PLGA 3D scaffold.

As shown in Fig. 1, we designed and prepared a 3D-printed PLGA scaffold in our lab according to the procedure described in the literature.^31^ Then, we soaked the 3D-printed PLGA scaffolds (PLGA) in dopamine solution (2 mg mL^−1^ in 10 mM Tris·HCl, pH 8.0) and placed them on an oscillator for 2 h at ambient temperature. Next, we washed the pDA-coated 3D PLGA scaffold (pDA-PLGA) with deionized water three times to remove unattached dopamine molecules.

### 2.3 BMP-2 and ponericin G1 grafting onto pDA-coated 3D PLGA scaffolds

We placed the pDA-coated 3D PLGA scaffolds in a 24-well plate, then introduced 1 mL of BMP-2 (200 ng mL^−1^ in phosphate-buffered saline (PBS)) and ponericin G1 (250 μg mL^−1^ in PBS) to the PLGA and pDA-PLGA scaffolds. Then, we incubated the scaffolds in the BMP-2 solution for 2 h at room temperature in a shaker to prepare BMP-2-immobilized 3D PLGA scaffolds (pDA-PLGA/BMP-2), ponericin G1-immobilized 3D PLGA scaffolds (pDA-PLGA/ponericin G1) and BMP-2/ponericin G1-immobilized 3D PLGA scaffolds (pDA-PLGA/ponericin G1/BMP-2). Next, we washed the scaffolds with distilled water three times to remove unattached peptides. The BMP-2-immobilized 3D PLGA scaffolds were then incubated with a primary antibody (rabbit polyclonal antibody anti-BMP-2) for 2 h at room temperature, and then washed with PBS three times for 5 min each time. The next step was incubation with Alexa Fluor 488 labeled goat anti rabbit IgG antibody (1 : 2000 dilution) for 2 h at room temperature in the dark. To compare their binding abilities, we immersed the pDA-PLGA and PLGA scaffolds in FITC-ponericin G1 (250 μg mL^−1^ in PBS) for 2 h at room temperature. The scaffolds were then washed with distilled water three times. All these scaffolds were then imaged by a fluorescent imaging system (CRI Maestro 500FL) to observe the binding activity of BMP-2 and FITC-ponericin G1 on the pDA-PLGA and PLGA 3D scaffolds. We used a fixed exposure time at all-time points and performed a semi-quantitative comparison of the different scaffolds using commercial software (Maestro™ 2.4).

### 2.4 Uptake and release of BMP-2 and ponericin G1

We immersed the pDA-PLGA and PLGA 3D scaffolds in BMP-2 (200 ng mL^−1^) and ponericin G1 (250 μg mL^−1^) solutions and incubated them for 2 h at room temperature. After incubation, we performed the enzyme-linked immunosorbent assay to measure the concentration of BMP-2 in the solution and measured the concentration of ponericin G1 at OD_280_. The extinction coefficient of ponericin G1, as calculated by the ExPASy tool (https://web.expasy.org/cgi-bin/protparam/protparam), was 5.134 (1 mg mL^−1^). We calculated the amount of immobilized BMP-2 and ponericin G1 on the scaffolds as the difference in the amounts of BMP-2 in the original and final solutions. After absorbing the BMP-2 and ponericin G1, we incubated the scaffolds in 2.0 mL of PBS at 37 °C while stirring at 60 rpm. At specified time intervals, we collected 0.2 mL of the supernatant and measured the protein content using the above method. To obtain the release profiles, we plotted the percentage of the cumulative content of released protein against time. All experiments were performed in triplicate.

### 2.5 Characterization of 3D printed scaffolds

Using a SEM, XL30 FEG, Philips, we examined the morphology of different 3D-printed scaffolds. To analyse surface hydrophobicity, we measured the static water contact angle with a contact angle analyser (VCA 2000, AST). We measured scaffold porosity using a micro-CT (SkyScan N.V., Kontich, Belgium) and tested the mechanical properties of the matrices using a universal mechanical testing machine (Instron 1121, UK).

### 2.6 Antibacterial activity assay

To test the antimicrobial activities of the 3D scaffolds, we used *S. aureus* and *E. coli* as model microorganisms. To investigate the inhibitory effect of the different 3D scaffolds, we employed a disc diffusion method, whereby we first spread 100 mL of 10^5^ CFU *S. aureus* or *E. coli* suspension on a nutrient agar plate, and placed each disc to be tested on agar for 16 h incubation at 37 °C. Then, we pressed the different 3D scaffolds into the discs and incubated them for 24 h at 37 °C, after which we examined the plates for the presence of inhibition zones around the samples. For the different 3D scaffolds, to determine their relative bacteria killing efficiencies, we mixed the scaffolds with 5 mL of *S. aureus* and *E. coli* in an LB culture medium (4.0 × 10^4^ bacteria per mL), respectively, and cultured them for 24 h. The bacterial growth was measured as turbidity at an optical density at OD 600 nm, using on a Microplate Reader (Infinite M200, TECAN) and a 96-well cell culture plate (Sigma). We used a bacterial suspension without 3D scaffolds as the control, and obtained all results in triplicate.

### 2.7 Cell adhesion and proliferation

Cell experiments were conducted using MC3T3-E1 mouse cells. Each scaffold was sterilized by ultraviolet rays (UV) for 30 min followed by the seeding of 2 × 10^4^ MC3T3-E1 cells on each scaffold in a humidified incubator at 37 °C and 5% CO_2_. After 1 to 7 days in culture, WST-8 solution (10% v/v in medium) (CCK-8) was added to each well. After 2 h of incubation, 100 μL of the solution from each well was moved to a new 96-well plate. We measured the absorbance value at 450 nm on a multifunction microplate scanner (Tecan Infinite M 200) and calculated the cell proliferation rate. To determine the degree of cell attachment, we visualized the MC3T3-E1 cells on the surfaces of different 3D scaffolds using only the ‘Live’ kit (green) from a Live/Dead assay kit (Biotium, USA). At a predesignated time, we washed these cell-containing scaffolds with PBS three times and treated them with calcein AM (2 μM) and propidium iodide (4 μM) for 30 min at room temperature. Finally, we examined the cell/scaffold samples under a fluorescence microscope (TE2000-U, Nikon).

### 2.8 Calcium deposition

We performed Alizarin Red-S (ARS) staining to assess the calcium deposition by the MC3T3-E1 cells that had been incubated on the substrates for 21 days. Briefly, we washed the scaffolds with the attached cells three times with PBS (pH 7.4) and fixed them with 4% glutaraldehyde solution for 15 min. Subsequently, we stained the samples in 2% ARS for 30 min, after which we washed them three times in PBS and examined them through a light microscope. We measured the calcium using a cetylpyridinium chloride (CPC) treatment. After washing the ARS-stained scaffolds samples with deionized water, we treated them with 1 mL of 10% CPC solution for 1 h and measured the absorbance of the solution at 540 nm in a full wavelength reader.

### 2.9 Expression of osteogenesis-related genes

After incubating the MC3T3-E1 cells on various 3D scaffolds for 7 days, we explored the expression of osteogenic genes *via* a quantitative real-time polymerase chain reaction (qRT-PCR). Briefly, we isolated RNA from the samples in 24-well plates using TRIzol (Invitrogen, USA), then synthesized cDNA using the PrimeScript RT reagent kit and primers for the osteosis-related genes (Runx-2, OPN and OCN), as described in a previous study (Table 1). We used GAPDH as the internal reference gene, and performed gene amplification according to the SYBR Premix EX *Taq* protocol using a real-time PCR instrument (Stratagene 3005p). The qPCR amplification was performed as follows: initial denaturation at 95 °C for 10 min, followed by 40 cycles at 95 °C for 30 s, 58 °C for 1 min and 72 °C for 1 min. We calculated the relative gene expression using the Stratagene MxPro software v4.01 system and reported the results as relative gene expression. All experiments were done in triplicate to obtain the average data values.

**Table 1:** List of genes and primer nucleotide sequences

Gene annotation	Forward primer sequence	Reverse primer sequence
OPN	TCAGGACAACAACGGAAAGGG	GGAACTTGCTTGACTATCGATCAC
	R: GGAACTTGCTTGACTATCGATCAC	24
OCN	AAGCAGGAGGGCAATAAGGT	TTTGTAGGCGGTCTTCAAGC
	GCCGGGAATGATGAGAACTA	GGACCGTCCACTGTCACTTT
Runx2	R: GGTCAGTCAGTGCCTTTCCTC	21
GAPDH	AACTTTGGCATTGTGGAAGG	ACACATTGGGGGTAGGAACA
	R: CAACCTGGTCCTCAGTGTAGC	21
	P: CGTGCCGCCTGGAGAAACCTGCC	23

### 2.10 Statistical analysis

The data were analysed using Origin 8.0 and are presented as the mean ± standard deviation. For the analysis of multiple groups, we evaluated statistical differences by one-way analysis of variance (ANOVA, Origin 8.0). We considered a *p*-value of <0.05 to be statistically significant.

## 3. Results and discussion

### 3.1 Scaffold morphology and structure

**Fig. 2:**
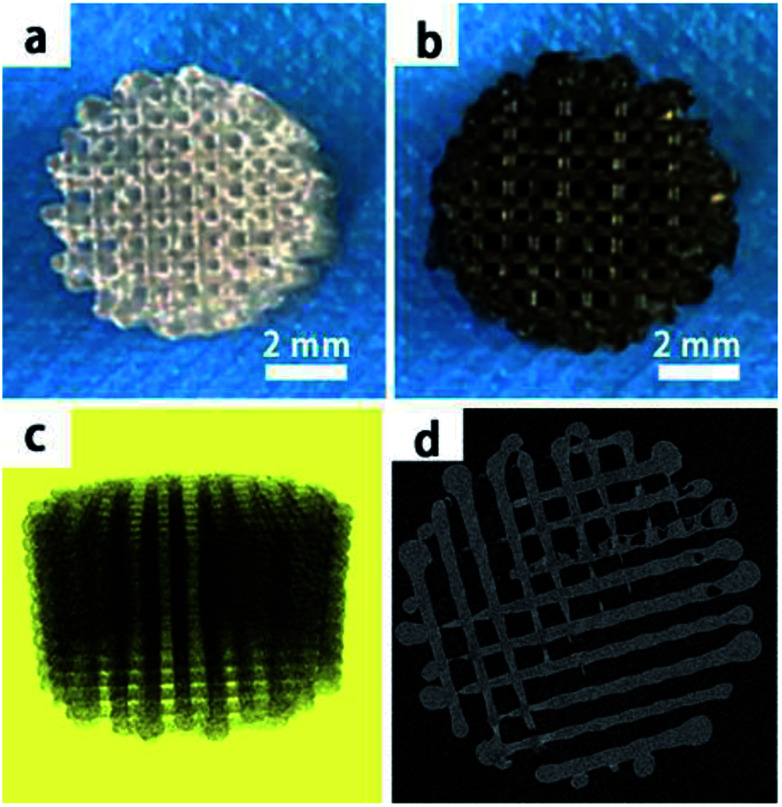
The macroscopic view of PLGA and pDA-PLGA 3D printed scaffolds (a and b), micro-CT micrographs of PLGA 3D printed scaffolds (c and d).

**Fig. 3:**
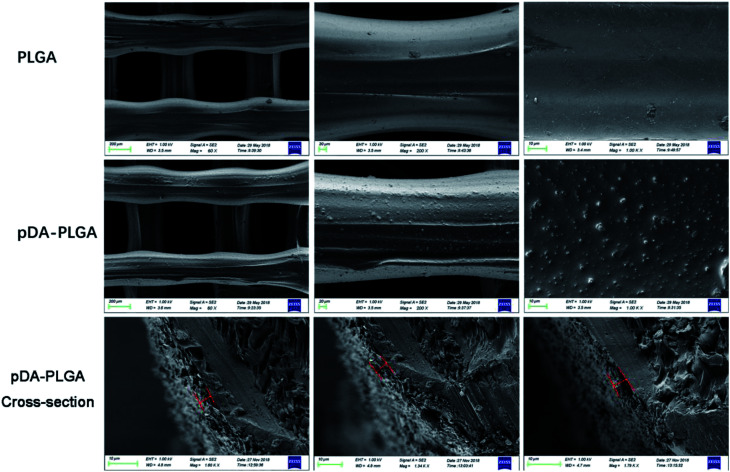
SEM images of PLGA, pDA-PLGA and pDA-PLGA cross-section 3D printed scaffolds. (The red line indicated pDA coating thickness).

In this study, we mainly focused on preparing functionalized 3D-printed PLGA scaffolds with a pDA coating to realize the affinity adsorption of BMP-2 and ponericin G1. First, we immersed the 3D-printed scaffolds in an alkaline solution of dopamine for 12 h to form an adherent pDA coating. Subsequently, the modified scaffold was used as a support to immobilize the mixture of BMP-2 and ponericin G1. The 3D-printed PLGA scaffolds were fabricated using a bioplotting system consisting of a heating tube, print nozzle, nitrogen pressure system, an x–y–z stage and a computer system. Fig. 2 shows micro-CT images of the different 3D-printed scaffolds, in which we can see that the micropores exhibit highly connected porosity, which is in accordance with the design. After the dopamine solution treatment, the pDA-PLGA scaffolds are easily distinguished by the colour change of the scaffolds (black) due to pDA surface modification. Furthermore, the SEM micrographs in Fig. 3 show that the pure PLGA scaffolds have a smooth surface. After the dopamine solution treatment, the surface roughness of PLGA scaffolds increased whereas the 3D configuration of the scaffolds were retained. The diameters of the micropores ranged between 468.9 μm and 491.3 μm. As shown in the cross-section images of the pDA-PLGA, the thickness of the pDA on 2 mg mL^−1^ of the pDA-PLGA scaffold was between 3.66 μm and 5.18 μm. The same results are also reported in the literature and the pDA coating might alter the surface performances of the polymer matrix, including its surface roughness and hydrophilicity.^32^ By SEM observation, the surface coatings can be clearly observed on PLGA scaffolds.

### 3.2 Mechanical property analyses

**Fig. 4:**
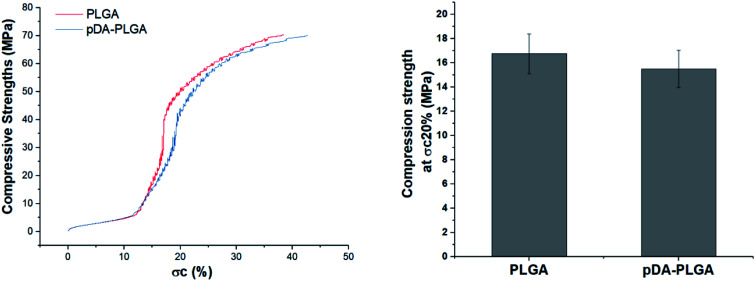
Compressive strength of the PLGA and pDA-PLGA 3D scaffolds were obtained under a cross-head speed of 2 mm min^−1^. *n* = 3.

The microarchitecture and porosity of 3D scaffolds, including the pore size, distribution and connectivity, play crucial roles in recreating a tissue-like cell mass. Porous scaffolds are designed to provide mechanical support until the regenerative tissue or organ is structurally stabilized. Therefore, maintaining the mechanical properties of 3D-printed scaffolds is crucial in biomedical applications. Ideally, their mechanical properties are similar to those of the supported tissue. To evaluate the mechanical strength of the different 3D printed scaffolds, Fig. 4 shows the compressive strengths of the PLGA and pDA-PLGA scaffolds. For the PLGA scaffolds, the compressive strength at *σ*_c_ (20%) reached 16.74 ± 1.62 MPa. The pDA-PLGA scaffolds exhibited similar compression strength values, so we can conclude that the pDA coating did not change this mechanical property of 3D PLGA scaffolds. The above results show that the compressive strength of PLGA and pDA-PLGA scaffolds are similar to that of natural bone, which is beneficial to the biomimetic design of bone-tissue scaffolds.

### 3.3 Contact angle analysis

**Fig. 5:**
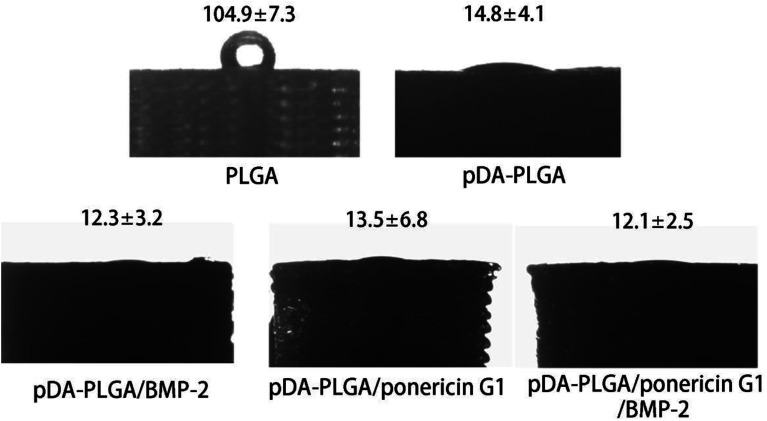
Water contact angle measurement for the PLGA, pDA-PLGA, pDA-PLGA/BMP-2, pDA-PLGA/ponericin G1 and pDA-PLGA/BMP-2/ponericin G1 scaffolds.

The hydrophilicity of materials plays an important role in their interaction with cells. Improvement in the hydrophilicity of the material surface can enhance the cellular behaviours, including the cell adherence, proliferation and differentiation. To evaluate whether the hydrophilicity of the 3D PLGA scaffold is improved by pDA coating, we measured the water contact angles on the surfaces of the PLGA and pDA-PLGA scaffolds. As shown in Fig. 5, the water contact angle was 104.9 ± 7.3° for the PLGA scaffolds and 14.8 ± 4.1° for the pDA-PLGA scaffolds, which indicates a significant increase in wettability in the presence of pDA coating on the substrates. The water contact angle of pDA-PLGA/BMP-2, pDA-PLGA/ponericin G1 and pDA-PLGA/BMP-2/ponericin G1 were 12.3 ± 3.2°, 13.5 ± 6.8° and 12.1 ± 2.5°, respectively. After the pDA-PLGA surface had been coated with BMP-2 and ponericin G1, the hydrophilicity of the 3D scaffold was slightly but not significantly improved.

The above contact angle measurement results indicate that the hydrophilicity of 3D-printed scaffolds was improved by the pDA/BMP-2/ponericin G1 coating. Therefore, we can conclude that the proliferation rate of cells can be improved if they are grown on materials with a hydrophilic surface.^33,34^

### 3.4 Binding of BMP-2 on 3D PLGA scaffolds

**Fig. 6:**
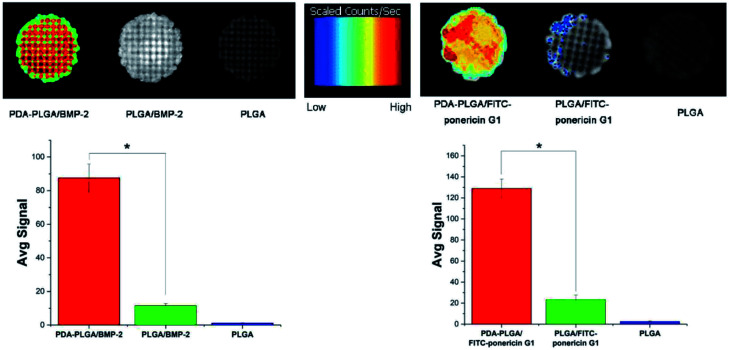
BMP-2 and FITC-ponericin G1 binding ability of PLGA and pDA-PLGA scaffolds were detected by immunofluorescence. (*n* = 3, *p < 0.05).

Next, we determined the binding efficiency of BMP-2 on pDA-PLGA scaffolds and compared the results with those of PLGA scaffolds using an immunofluorescence assay with a monoclonal anti-BMP-2 antibody and a secondary Alexa-Fluor 488-labeled anti-mouse IgG antibody. Fig. 6 shows that the fluorescence signal of the pDA-PLGA/BMP-2 scaffold was higher than those of the PLGA/BMP-2 and pure PLGA scaffolds. These results indicate that the amount of BMP-2 on the surface of the pDA-PLGA scaffold was higher than that on PLGA scaffolds. The average signal of BMP-2 on pDA-PLGA scaffolds was 8.35-fold higher than that on PLGA. There are two reasons for this: first, pDA-PLGA scaffolds have a rougher surface morphology; second, many different interactions occur on the pDA layer, such as the reaction of catechols with thiols and amines.^24,35^ Previous studies have demonstrated that a dopamine (DA)-based method, *i.e.* the self-polymerization of dopamine, can be used to immobilize biomolecules on a wide range of natural and synthetic inorganic/organic materials.^36,37^ The above results suggest that pDA-PLGA scaffolds have good affinity with protein and may be an effective carrier for BMP-2 delivery. We used the same method to detect the binding efficiency of pDA-PLGA and PLGA scaffolds with ponericin G1. As shown in Fig. 6, pDA-PLGA scaffolds showed a stronger binding capacity with ponericin G1 than the PLGA scaffolds. The average signal of pDA-PLGA was 5.53 times that of PLGA. The above results show that pDA coatings on PLGA scaffolds can effectively bind to ponericin G1, and that increased loading of ponericin G1 on pDA-PLGA scaffolds will enhance the antibacterial activity of the scaffolds.

### 3.5 Adsorption and release of BMP-2 and ponericin G1

**Fig. 7:**
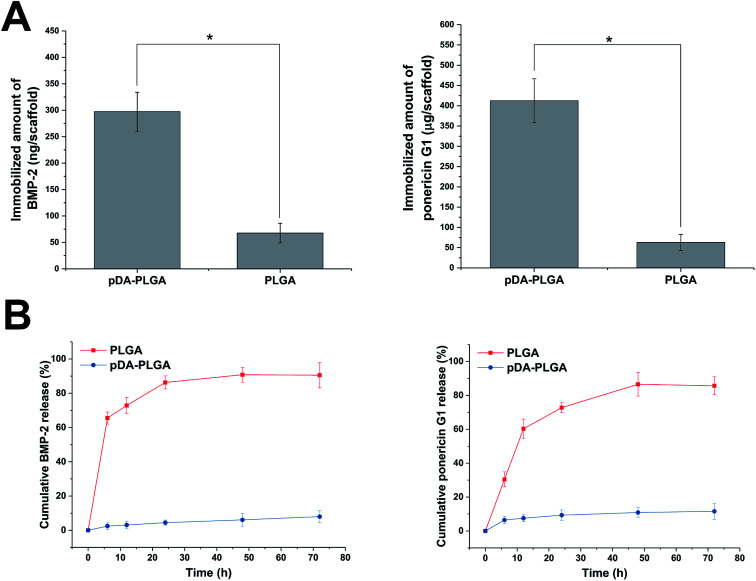
(A) Quantification of BMP-2 and ponericin G1 immobilized on the 3D-printed scaffolds (PLGA and pDA-PLGA). (B) Kinetics of BMP-2 and ponericin G1 release from the 3D-printed scaffolds (PLGA and pDA-PLGA) at different time periods. (*n* = 3, *p < 0.05).

As shown in Fig. 7A, BMP-2 and ponericin G1 were tightly immobilized on the surfaces of the pDA-coated scaffolds. The amount of immobilized BMP-2 on the pDA-coated scaffolds was about 297 ng per scaffold. The efficiency of BMP-2 immobilization on pDA-coated scaffolds was about 9.70 times that of the uncoated scaffolds. The amount of immobilized ponericin G1 on pDA-PLGA was about 412 μg per scaffold. The absorptive capacity of ponericin G1 on pDA-PLGA was about 6.54 times that of the PLGA scaffold. Investigation of the BMP-2 and ponericin G1 release profiles from pDA-PLGA and PLGA scaffolds for up to 72 h (Fig. 7B) revealed that about 86.31% of BMP-2 and 76.75% of ponericin G1 were burst released from the PLGA scaffolds during the first 24 h and only 9.38% of BMP-2 and 16.43% of ponericin G1 were released from the pDA-PLGA scaffolds during 72 h of the experiment. These results clearly show that BMP-2 and ponericin G1 had been stably immobilized on the surfaces of the pDA-coated scaffolds, which effectively reduced the burst release of this growth factor and polypeptide. Also, the immobilized growth factor and polypeptide maintained a sustained release rate compared to that of the control group. Therefore, since growth factors and polypeptides can be enabled to serve biological functions for longer periods of time, this reduces the risk associated with the need for the continuous use of large amounts of these materials.

### 3.6 Antibacterial activity

**Fig. 8:**
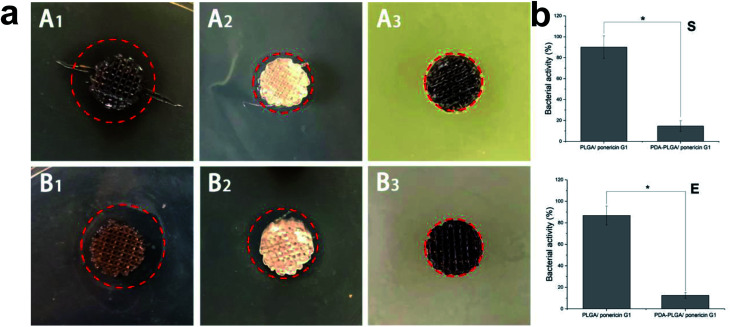
(a) Inhibition zones study of the PLGA/ponericin G1 (A_2_ and B_2_), pDA-PLGA/ponericin G1 (A_1_ and B_1_) and pDA-PLGA (A_3_ and B_3_) against *E. coli* (E) (B_1_, B_2_ and B_3_) and *S. aureus* (S) (A_1_, A_2_ and A_3_), (b) relatively antibacterial activity of E and S, after 24 h of culture at 37 °C. (*n* = 3, *p < 0.05).

Lack of antibacterial properties is a serious obstacle that limits the biomedical application of biopolymers, including PLGA. Therefore, the use of topical antimicrobial agents is necessary to deal with avascular necrotic tissues in wounds, which are rich in exudations and proteins. In recent years, many studies have demonstrated the biocidal activity of ponericin G1 against a broad spectrum of bacteria, and its ability to be manually synthesized without raising any immunological concerns.^30^ Thus, in this study, we used the pDA-modified 3D PLGA scaffold as a support to immobilize ponericin G1. To verify the antibacterial activity of ponericin-G1-immobilized 3D PLGA scaffolds, we investigated both *E. coli* and *S. aureus* in our experiments. Fig. 8a shows the disc diffusion results of the PLGA/ponericin G1, pDA-PLGA ponericin G1 and pDA-PLGA scaffolds against *E. coli* (Gram-negative) and *S. aureus* (Gram-positive) bacteria. We observed an obvious inhibition zone on the PLGA/ponericin G1 and pDA-PLGA ponericin G1 samples for both *E. coli* and *S. aureus* after 24 h of culture on nutrient agar, which indicates the antibacterial activity of ponericin G1. The pDA-PLGA scaffold showed no inhibition zone for either *E. coli* or *S. aureus* after 24 h of culture. However, we found the growth of both *E. coli* and *S. aureus* to be more inhibited by the pDA-PLGA/ponericin G1 scaffold than by the PLGA/ponericin G1 scaffolds. As shown in Fig. 8b, the pDA-PLGA/ponericin G1 scaffold has better antibacterial ability with respect to both *E. coli* and *S. aureus*. Therefore, we deduced that the high protein affinity of the pDA-PLGA scaffolds results in a higher immobilization efficiency of ponericin G1 than that of pure PLGA scaffolds, and that immobilized ponericin G1 retains its bioactivity for a longer time, which affords these 3D scaffolds long-term antibacterial activity.

### 3.7 MC3T3-E1 cell growth on 3D PLGA scaffolds

**Fig. 9:**
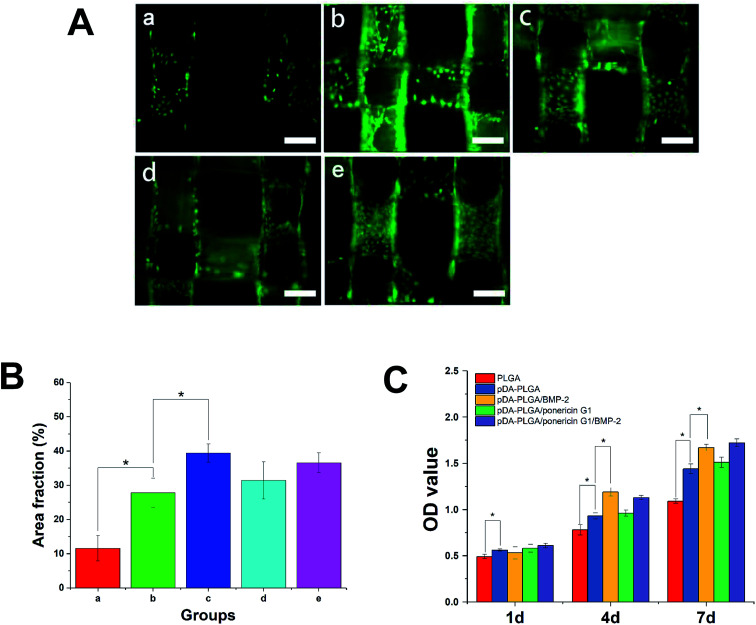
(A) Fluorescent staining observation of MC3T3-E1 cells cultured on different 3D printed scaffolds for 3 days. (a) PLGA; (b) pDA-PLGA; (c) pDA-PLGA/BMP-2; (d) pDA-PLGA/ponericin G1 (e) pDA-PLGA/ponericin G1/BMP-2. All scale bar lengths are 500 μm. (B) Area fraction of MC3T3-E1 cells cultured on different 3D printed scaffolds. (C) Proliferation of MC3T3-E1 cells cultured on different 3D printed scaffolds 1 to 7 days *in vitro*. *p < 0.05 compared to the other group, *n* = 3.

Initial cell adhesion is usually responsible for cellular functions and eventual tissue integration, whereas cell proliferation is closely correlated to the amount of new bone formed. Therefore, first, we used calcein AM fluorescent staining to investigate cell adhesion with BMP-2 or/and ponericin G1 immobilization *via* pDA coating. As shown in Fig. 9A, after culturing for 3 days, the number of cells on the pDA-PLGA scaffolds was greater than those on the PLGA scaffolds. After the BMP-2 was immobilized, the densities of cells on the pDA-PLGA/BMP-2 and pDA-PLGA/ponericin G1/BMP-2 scaffolds were higher than those on the other scaffolds. NIH Image ImageJ analysis (Fig. 9B) results showed that the cell area percentage in the pDA-PLGA group was higher than that in the PLGA groups. After the BMP-2 or/and ponericin G1 was immobilized, the cell area percentage in the BMP-2-immobilized group was significantly higher than in other groups, but there was no significant change in the ponericin G1-immobilized group.

The above results indicate that better cell adhesion and positive cellular interaction occurred in the presence of the supporting structure of the BMP-2-immobilized 3D scaffold of PLGA. Furthermore, after ponericin G1 was immobilized, we found no obvious effect on cell adhesion; the cells maintained a relatively healthy morphology. This suggests that ponericin G1 has excellent antibacterial activity as well as good cell compatibility.

Next, we investigated the MC3T3-E1 cell proliferation on the 3D PLGA scaffold by performing a CCK-8 assay. As shown in Fig. 9C, compared to the PLGA scaffolds, the cells showed greater proliferation on the pDA-PLGA porous scaffolds at 4 days and 7 days. Therefore, we can conclude that pDA-coated 3D PLGA scaffolds showed positive trends in promoting cell proliferation. This result is consistent with reports in the literature regarding the significant effects of pDA-coated substrates in stimulating cell proliferation.^23,24,31^ Furthermore, we found a statistical difference in cell proliferation between the pure pDA-PLGA scaffolds and BMP-2-immobilized 3D PLGA scaffolds, which suggests that BMP-2 can promote cell proliferation. Researchers have reported that the expression of integrin β1, fibronectin and integrin a5, which are promoted by BMP-2, is required for cell spreading, adhesion and proliferation.^38,39^ Ponericin G1 showed no killing effect toward MC3T3-E1 cells. These results are consistent with the cell adhesion results.

### 3.8 Cell mineralization

**Fig. 10:**
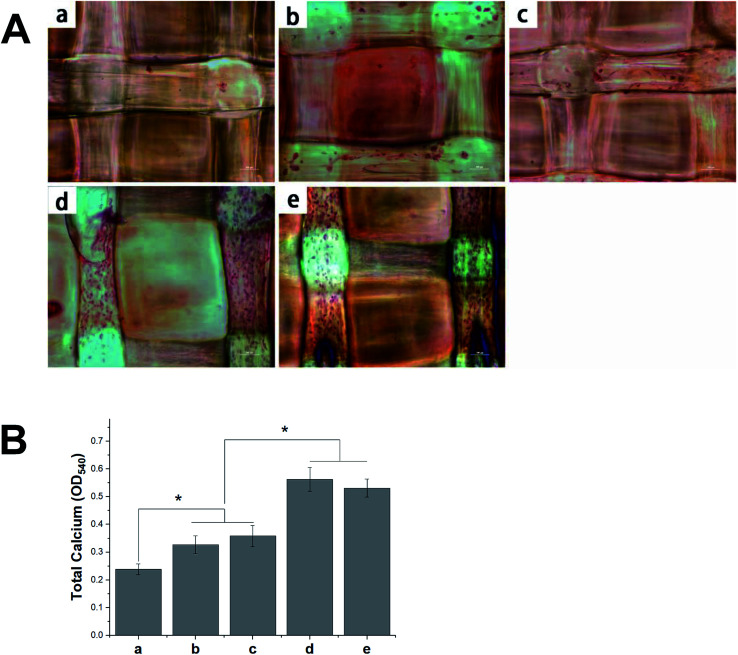
(A) Alizarin Red staining of MC3T3-E1 cells cultured on PLGA (a), pDA-PLGA (b), pDA-PLGA/ponericin G1 (c), pDA-PLGA/BMP-2 (d), and pDA-PLGA/BMP-2/ponericin G1 scaffolds (e). (B) The corresponding quantitative evaluation of calcium content mineral deposition in MC3T3-E1 cells cultured for three weeks.

The deposition of calcium phosphate in the ECM is indicative of osteogenesis and is used as a marker for bone regeneration. In this study, we performed ARS staining to visualize the accumulated calcium deposition, as shown in Fig. 10A. We found the MC3T3-E1 cells that had been seeded on different 3D scaffolds to show both material- and time-dependent mineralization. The ARS produced slight reddish dots on the pDA-PLGA scaffolds, but almost no positive stains on the pure PLGA scaffolds. This indicates that the pDA coating could induce osteogenic differentiation in the MC3T3-E1 cells. Many studies have reported that the universal adhesive property of the catechol group in pDA coatings might play a certain role in the formation of HA and facilitate cell mineralization.^40,41^ After BMP-2 had been immobilized, an increase in calcium staining was observed on the pDA-PLGA/BMP-2 scaffolds, as well as the generation of newly formed inter-connected cells between adjacent columns. This indicates that the bioactivity of BMP-2 was retained during the pDA coating process, and the release of BMP-2 showed improved cell osteogenesis differentiation. Several studies have reported the excellent bone induction activity of BMP-2 to be largely due to the mineralization effect of BMP-2 on cells.^42,43^ Interestingly, after the ponericin G1 had been immobilized, we observed slightly enhanced staining on day 21 in the ponericin-G1-immobilized scaffolds compared to the pure pDA-PLGA scaffolds. As a polypeptide, we speculated that ponericin G1 can improve the wettability of 3D scaffolds and provide some functional groups that can improve the nucleation of the HA.

As shown in Fig. 10B, to evaluate the calcium-rich mineral deposits of the MC3T3-E1 cells, we performed a quantitative assessment of the cell mineralization after extracting ARS with 10% CPC. We found the calcium content in the MC3T3-E1 cells on the pDA-PLGA and ponericin-G1-immobilized 3D scaffolds to be significantly higher than that of cells growing on the PLGA 3D scaffold. The 3D scaffolds incorporating BMP-2 exhibited the highest calcium content, which indicates enhanced differentiation of the MC3T3-E1 cells. The quantitative assessment of mineral deposition showed the same trend as the osteogenic protein expression of OCN, which indicates that the immobilization of BMP-2 and ponericin G1 *via* a pDA coating on PLGA 3D scaffolds can effectively promote the osteogenic differentiation of MC3T3-E1 cells.

### 3.9 Quantitative real-time polymerase chain reaction

**Fig. 11:**
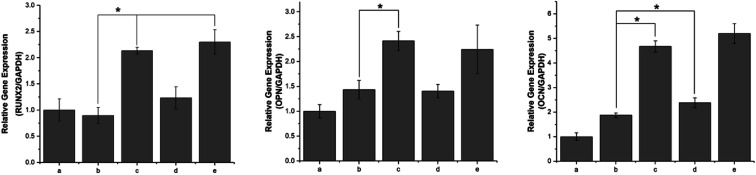
The qRT-PCR analysis for Runx2, OPN and OCN expression by MC3T3-E1 cells cultured on PLGA (a), pDA-PLGA (b), pDA-PLGA/BMP-2 (c), pDA-PLGA/ponericin G1 (d) and pDA-PLGA/BMP-2/ponericin G1 (e) for 7 days. * Indicates significant differences (*P* < 0.05), *n* = 3.

During the osteogenesis differentiation of cells, some key cytokines and functional proteins are regularly expressed. In this study, the selected mRNA expressions corresponding to specific genes were Runx2, OPN and OCN. RUNX2 is a differentiation marker observed at the early stage of differentiation, OPN is observed at the middle/late stage and OCN is observed at the late stage of differentiation.^44^ As shown in Fig. 11, compared with the unmodified PLGA scaffold, the pDA-PLGA scaffold groups show no significant increase in the gene expression of Runx2 in the MC3T3-E1 cells after 7 days of culture. However, we observed an up-regulation in the OPN and OCN gene expressions in the pDA-PLGA scaffolds after 7 days of culture. OPN and OCN are constituents of the bone matrix and have a high affinity for hydroxyapatite. Moreover, they play important roles in bone mineralization and calcification processes. Thus, these results indicate that the pDA coating played a more important role in the late stage of osteogenesis differentiation. When BMP-2 had been immobilized on the surface of the pDA-PLGA scaffolds, the Runx2, OPN and OCN expressions were further enhanced. This set of data confirm that the exposure of BMP-2 on pDA-PLGA scaffolds can further promote the osteogenic differentiation of MC3T3-E1 cells as compared to other 3D scaffolds. This may be attributed to the improved BMP-2 binding on the surface of the 3D scaffold surface, which results in 3D scaffolds having long-term osteoconductivity. However, after ponericin G1 had been immobilized, we found no significant increase in the Runx2 or OPN expressions and only a slight increase in the OCN expression on the ponericin-G1-immobilized 3D PLGA scaffolds. Therefore, we speculate that ponericin G1 plays little role in the osteogenesis differentiation of MC3T3-E1 cells. The above results demonstrate that BMP-2-and-ponericin-G1-immobilized pDA-PLGA 3D scaffolds can significantly enhance the osteodifferentiation of MC3T3-E1 cells. Combined with the results regarding antibacterial activity, cell growth, calcium deposition and osteogenesis-related gene expression, bioactive peptide immobilization *via* a pDA coating on PLGA 3D scaffolds seem to offer an optimal strategy for the fabrication of ideal bone-tissue-engineering scaffolds.

The healing of bone defects is a critical and complex biological process after trauma. Natural bone development and repair are orchestrated by a cascade of numerous biologically active proteins. In clinical practice, to prevent infection or enhance bone formation, patients are often prescribed oral or injected medications and bioactive peptides *via* systemic drug delivery. In addition to the above, in recent years, numerous research groups have added drugs or bioactive molecules to tissue-engineering scaffolds for topical administration purposes.^15,16^ To ensure the long-term biological activity of a drug or bioactive peptide on the surface of scaffolds, the immobilization method has attracted much attention as a new drug delivery method. Using this approach, the inherent properties of polymer scaffolds can be retained while allowing sustained local drug delivery. In this study, we modified 3D PLGA scaffolds with pDA to produce pDA-PLGA scaffolds, and we immobilized BMP-2 and ponericin G1 on the surface of the scaffolds with a catecholamine polymer. In our study, we found the pDA coating to obviously improve the surface hydrophilicity of 3D PLGA scaffolds and further enhance their biological activity. This result indicates that the introduction of abundant hydrophilic groups such as –OH, –COOH and –NH_2_ in the pDA layer brought hydrophilicity to otherwise hydrophobic PLGA porous scaffolds. More importantly, the binding efficiency of BMP-2 at the surface of pDA-coated PLGA scaffolds was greater than that of pure PLGA scaffolds, which not only reduces the amount of BMP-2 required, but also provides the 3D scaffolds with long-term osteoconductivity. Furthermore, in our antibacterial investigation, the immobilization of ponericin G1 *via* the pDA coating showed an effective antibacterial activity as high as 57.89% to *S. aureus* and 67.34% to *E. coli*, and the antibacterial activity of pDA-PLGA/ponericin G1 scaffolds were higher than those of the PLGA/ponericin G1 scaffolds. This bacterial inhibition can be attributed to the higher protein adsorption capacities of the pDA coating. According to previous reports,^41^ proteins can be immobilized on a PDA layer by amines *via* Michael addition or Schiff base reactions to achieve greater protein adsorption. Covalent conjugation might account for the higher levels of protein adsorption to the pDA coatings as compared to pure PLGA scaffolds. In our i*n vitro* experiment, the results indicated that cell adhesion and proliferation were increased by the pDA coating due to the improved surface properties, and the osteogenic gene expression of MC3T3-E1 cells were obviously increased by the incorporation of BMP-2. Many biological molecules have been successfully grafted onto the pDA-modified surfaces of various substances and show good biological activities, such as trypsin, hyaluronic acid and DNA.^45–47^ This strategy of creating a functional organic adlayer requires only two factors—simple ingredients and mild reaction conditions. The surface immobilization of BMP-2 *via* a PDA coating can not only reduce the amount of BMP-2 required, but also provide the composites with long-term osteoconductivity. Furthermore, we found ponericin-G1-immobilized 3D PLGA scaffolds to have an excellent antibacterial activity and no effect on the bioactivity of the 3D scaffolds. The application of antimicrobial peptides is of interest due to their wide range of activity against Gram-positive and Gram-negative bacteria, as well as their low onset of bacterial resistance. Previous research has found that ponericin G1 does not affect cell activity and is able to inhibit bacteria attachment.^30^ Our results showed that the antibacterial activity of ponericin G1 is retained in prepared 3D scaffolds and could be applied as an excellent antibacterial implant. Among all the 3D scaffolds, the pDA-PLGA/BMP-2/ponericin G1 group showed the highest bone repair capacity, which can be attributed to the synergistic effects of the antibacterial activity and excellent bioactivity. In summary, the pDA-assisted immobilization of bioactive peptides is a promising method for modifying the surface of 3D scaffolds for bone regeneration. However, ponericin G1 is regarded as a foreign chemical and the long-term effects of its stability and toxicity during the retention of ponericin G1 in the organism should be considered when applied in bone defect repair. In future work, an *in vivo* study of the pDA-PLGA/BMP-2/ponericin G1 is a necessary next step.

## 4. Conclusions

In this study, we prepared PLGA scaffolds using a 3D-printing method and then immobilized BMP-2 and ponericin G1 on the surface of the 3D PLGA scaffolds. We found the BMP-2/ponericin-G1-loaded 3D-printed PLGA scaffolds to maintain a stable porous 3D structure with no induced scaffold degradation, which promoted better adherence, proliferation and calcium deposition of MC3T3-E1 cells. Furthermore, a pDA coating on 3D PLGA scaffolds can effectively increase the binding sites of the porous scaffolds for BMP-2 and ponericin G1, which can effectively improve the bioactivity and antibacterial activity of 3D scaffolds. The results of our *in vitro* studies, including CCK-8 assay, fluorescence staining, calcium deposition and *q*-PCR analysis, indicated that a BMP-2/ponericin G1 coating on 3D PLGA scaffolds promoted improved adhesion, proliferation and osteogenesis differentiation of MC3T3-E1 cells compared to those of pure 3D PLGA scaffolds. All the results presented above prove that this bioactive peptide-immobilized 3D-printed PLGA scaffold shows great potential for the development of biodegradable bone implants.

## Conflicts of interest

There are no conflicts to declare.

## Supplementary Material
